# A Curious Novel Combination of *Nucleophosmin* (*NPM1*) Gene Mutations Leading to Aberrant Cytoplasmic Dislocation of *NPM1* in Acute Myeloid Leukemia (AML)

**DOI:** 10.3390/genes12091426

**Published:** 2021-09-16

**Authors:** Alessandra Venanzi, Roberta Rossi, Giovanni Martino, Ombretta Annibali, Giuseppe Avvisati, Maria Grazia Mameli, Paolo Sportoletti, Enrico Tiacci, Brunangelo Falini, Maria Paola Martelli

**Affiliations:** 1 Hematology and Clinical Immunology, Centro di Ricerche Emato-Oncologiche (CREO), University of Perugia, 06132 Perugia, Italy; alessandra.venanzi@gmail.com (A.V.); roberta.rossi78@virgilio.it (R.R.); paolo.sportoletti@unipg.it (P.S.); enrico.tiacci@unipg.it (E.T.); brunangelo.falini@unipg.it (B.F.); 2 Hematology Section, “Santa Maria della Misericordia” Hospital of Perugia, 06132 Perugia, Italy; mgrazia.mameli@ospedale.perugia.it; 3 Pathology Unit, Azienda Ospedaliera Santa Maria di Terni, University of Perugia, 05100 Terni, Italy; gio.martino@gmail.com; 4 Department of Pathology, AOU Cagliari, University of Cagliari, 09042 Cagliari, Italy; 5 Hematology and Stem Cell Transplant Unit, Campus Bio-Medico University of Rome, 00128 Rome, Italy; o.annibali@unicampus.it (O.A.); g.avvisati@unicampus.it (G.A.)

**Keywords:** acute myeloid leukemia, nucleophosmin, *NPM1*, nuclear export signal (NES), exportin-1/XPO1

## Abstract

*Nucleophosmin (NPM1)* mutations occurring in acute myeloid leukemia (AML) (about 50 so far identified) cluster almost exclusively in exon 12 and lead to common changes at the *NPM1* mutants C-terminus, i.e., loss of tryptophans 288 and 290 (or 290 alone) and creation of a new nuclear export signal (NES), at the bases of exportin-1(XPO1)-mediated aberrant cytoplasmic *NPM1*. Immunohistochemistry (IHC) detects cytoplasmic *NPM1* and is predictive of the molecular alteration. Besides IHC and molecular sequencing, Western blotting (WB) with anti-*NPM1* mutant specific antibodies is another approach to identify *NPM1*-mutated AML. Here, we show that among 382 AML cases with *NPM1* exon 12 mutations, one was not recognized by WB, and describe the discovery of a novel combination of two mutations involving exon 12. This appeared as a conventional mutation A with the known TCTG nucleotides insertion/duplication accompanied by a second event (i.e., an 8-nucleotide deletion occurring 15 nucleotides downstream of the TCTG insertion), resulting in a new C-terminal protein sequence. Strikingly, the sequence included a functional NES ensuring cytoplasmic relocation of the new mutant supporting the role of cytoplasmic *NPM1* as critical in AML leukemogenesis.

## 1. Introduction

Acute myeloid leukemia (AML) with *nucleophosmin* (*NPM1*) gene mutations is the most frequent AML among adult patients, accounting for about one third (27–35%) of all AML [1]. *NPM1*-mutated AML is characterized by cytoplasmic positivity for *NPM1* at immunohistochemical bone marrow trephine analysis, hence also the name NPMc^+^ AML [1]. *NPM1*-mutated AML displays peculiar biological and clinical features [1,2] that lead to its inclusion as a provisional entity first, and then as a definitive distinct entity in the World Health Organization (WHO) classification of the myeloid neoplasms [3].

*NPM1* is a multifunctional protein [4], essential for life [5], localized in the nucleolus, where it represents one of the most abundant proteins [6] and plays a key role in ribosome biogenesis, but also with shuttling activity between the cytoplasm and the nucleus, serving as molecular chaperone for both nucleic acids and proteins [4]. *NPM1* acts also as a nucleolar stress sensor, and as a regulator of p14ARF and p53 key tumor suppressors [4].

*NPM1* mutations are specific for AML [1] and highly stable during the course of the disease and at relapse [7], and never found in normal subjects [8], suggesting they are *gatekeeper* genetic lesions in AML. Indeed, *NPM1* mutation drives leukemia in mice when co-expressed with commonly co-occurring mutations [9]. *NPM1* mutations in AML are consistently heterozygous [1]. An amount of wild-type *NPM1* is always detectable in the nucleolus of *NPM1*-mutated leukemic cells, strongly suggesting that it may be required for their survival [10]. This is in keeping with the observation that the complete knockdown of *NPM1* alleles is embryonic lethal in mice [5].

*NPM1* shuttles between the nucleus and cytoplasm thank to specific protein domains including two nuclear localization signals (NLS), a C-terminal nucleolar localization signal (NoLS) and two intramolecular nuclear export signal (NES) motifs [2,11,12]. At the steady state, signals driving *NPM1* into the nucleolus/nucleus are prevalent over the export of the protein, which is mediated by the interaction of NES domains in *NPM1* with the exportin 1(XPO1) protein [12].

In *NPM1*-mutated AML, this equilibrium is altered as the consequence of the gene mutation that occurs almost exclusively in the last exon of the gene (exon 12) causing changes at the C-terminus end of the protein, including loss of the NoLS and acquisition of a new NES, resulting in an aberrant dislocation of *NPM1* in the cytoplasm of AML cells [1,13,14]. This also occurs with the rarest mutations not involving exon 12 (i.e., exon 5, 9 and 11) [15,16,17].

We have previously shown that cytoplasmic *NPM1* and its interaction with exportin 1(XPO1) are essential for the maintenance of leukemic phenotype [18].

Here, we describe an interesting new combination of *NPM1* mutations in exon 12, leading to a mutated amino acid sequence at the C-terminus end, with loss of the NoLS of the wild type protein and creation of a NES motif, as shown for all the other exon 12 mutations [2,13,14].

## 2. Materials and Methods

### 2.1. Patients’ Samples

**Figure 1:**
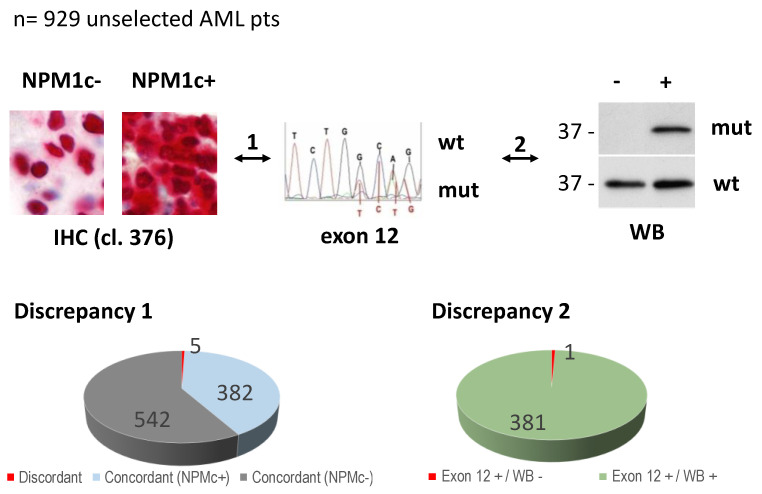
Study design and diagnostic discrepancies. Overall, 929 unselected AML patients were studied by multiple diagnostic approaches (i.e., immunohistochemistry, IHC; standard molecular analysis for *NPM1* exon 12, and Western blotting with specific anti-*NPM1* mutant antibody, WB). 1 indicates comparison between IHC and *NPM1* exon 12-molecular analysis. 2 indicates comparison between *NPM1* exon 12-molecular analysis and WB. wt: wild type (exon 12 wild type); mut: mutated (exon 12 mutation A and other non-A mutations). Discrepancy 1 indicates NPM1c+ at IHC and *NPM1* exon 12 wild-type (reported in [17]). Discrepancy 2 indicates NPM1c+ with mutation in exon 12 (Exon 12 +), but Western blotting (WB) negative with anti-*NPM1* mutant antibody, recognizing either *NPM1* mutant A and other previously described non-A mutants [20].

From 2005 to 2019, 929 AML patient samples referred to our center were studied by immunohistochemistry (IHC), Western blot (WB) with an anti-*NPM1* mutant antibody, and *NPM1* exon 12 sequencing, as routine diagnostic procedures, as previously reported [1,17,19,20,21] (Figure 1). The study was conducted in accordance with the Declaration of Helsinki, and informed consent was obtained from each patient to perform diagnostic examinations.

### 2.2. Immunohistochemical Analysis

Immunohistochemistry was performed on human paraffin embedded bone marrow trephine samples, as previously described [1,19]. Briefly, samples were fixed in B5 (Bio-Optica) for 2 h and decalcified in ethylenediaminetetraacetic acid (Osteodec; Bio-Optica, Milan, Italy) for 5 to 6 h. Antigen retrieval was carried out by microwaving in 0.1 mM ethylenediaminetetraacetic acid, pH 8.0. Cytoplasmic nucleophosmin was revealed using a mouse anti-*NPM1* monoclonal antibody, recognizing the N-terminal part of *NPM1* (mAb, clone 376, generated by B.F.) [1]. Notably, this is a pan-*NPM1* antibody able to recognize either *NPM1* wild-type or all the *NPM1* mutants [1,17,21]. Other antigens used for diagnosis included: nucleolin (C23; mAb MS-3; Santa Cruz Biotechnology, Dallas, TX, USA), myeloperoxidase (rabbit antimyeloperoxidase antibody; Dako, Glostrup, Denmark), linker for activation of T cells (LAT; Dako, Glostrup, Denmark); macrophage-restricted CD68 (mouse mAb PG-M1 generated by B.F.), CD34 (mouse mAb, clone QBEnd10, Dako, Glostrup, Denmark), and Bcl-2 (mouse mAb, clone 124, Dako, Glostrup, Denmark). The antibody/antigen interaction was revealed by the alkaline phosphatase anti-alkaline phosphatase (APAAP) technique.

### 2.3. Detection of NPM1 Mutant Protein by Western Blot Analysis

WB analysis for *NPM1* mutant protein was performed according to standard procedures, as previously described [20]. Lysates from 1−2 × 10^6^ cells from either peripheral blood or bone marrow of patients were used for analysis, whilst protein lysate from the human leukemic cell line OCI-AML3 (carrying *NPM1* mutation A) [22,23] was used as a positive control. The following antibodies were used: a rabbit polyclonal anti-*NPM1* mutant antibody (produced by B.F.), recognizing *NPM1* exon 12 mutants [20] and the-pan *NPM1* antibody, clone 376 [1]. After incubation with the appropriate secondary antibody, the signal was revealed by enhanced chemoluminescence, according to manufacturer’s instructions (Immobilon Crescendo Western HRP substrate, Millipore, Burlington, MA, USA).

### 2.4. Sanger Sequencing of NPM1

Genomic DNA and total RNA were extracted from bone marrow samples with AllPrep DNA/RNA mini kit (QIAGEN, Hilden, Germany). RNA was reverse-transcribed to generate cDNA for polymerase chain reaction (PCR) reaction using RT-kit plus (ELITech Group, Puteaux, France), following manufacturer’s instructions. cDNA was amplified using NPM-Forward primer: 5′-GAAAAAGCGCCAGTGAAGAAA-3′, and NPM-reverse primer: 5′-TACCGTGTTTGATAAATGTTGTCCAGG-3′. The PCR was performed using Platinum Taq DNA polymerase (Invitrogen, Waltham, MA, USA) (Tm = 60 °C; 38 cycles) and subjected to Sanger sequencing according to standard protocol, using ABI 3500 Genetic Analyzer (Applied Biosystems, Inc., Waltham, MA, USA). The raw data obtained from the sequencer were analyzed with FinchTV 1.4.0 (Geospiza, Seattle, WA, USA) in order to obtain the nucleotide sequence.

### 2.5. Targeted Deep Sequencing

Genomic DNA was quantified with High-Sensitivity (HS) and Broad-Range (BR) Qubit assay on a Qubit 2.0 fluorometer (Invitrogen) and was subjected either to deep targeted sequencing of *NPM1* gene to molecularly barcoded targeted sequencing of 42 genes recurrently mutated in myeloid neoplasms (QIAseq Targeted DNA Custom Panel–CDHS-31667Z-1041-QIAGEN) (Supplementary Table S1), using 35 ng of input DNA. Libraries, generated according to the manufacturer’s instructions, were sequenced on a Illumina MiSeq instrument 2 × 151 cycles for the myeloid panel, using MiSeq Reagent Kit v2 (Illumina, San Diego, CA, USA) (mean unique depth of coverage was 1508×). Mapping and variant calling was performed with QIAGEN smCounter algorithm v2 with default settings [24], and variant annotation was performed with Illlumina Variant Studio 3.0. Sequencing variants were then subjected to the further following filters, and retained only if: (i) they passed the default filters of the Qiagen software; (ii) they were predicted to change the gene coding sequence, or they involved the conserved splice-site (i.e., the 4 nucleotides surrounding the exon-intron junction); (iii) they were not present in the Exome Aggregation Consortium (ExAC) database of normal individuals at a population frequency ≥1% (as provided by Illumina Variant Studio 3.0); (iv) they were present at an allele frequency >2%; and (v) if consisting in insertions or deletions, they were not located in homopolymeric stretches of 5 nucleotides or longer.

### 2.6. Genomic DNA NPM1 Fragment Analysis

Genomic DNA was extracted with Maxwell (Promega, Madison, WI, USA), quantified using a ND-1000 apparatus (Nanodrop Technologies, Wilmington, DE, USA), and amplified with the NPM-Forward primer: FAM 5′-AGGACAGCCAGATATCAACTGTTAC-3′ and NPM-Reverse primer: 5′-AGTTAACTCTCTGGTGGTAGAATGAAA-3′. Reactions of 25 μL contained 10 ng of genomic DNA, primers (0.8 µM each), deoxynucleoside-5′-triphosphates (0.25 mM, each), MgCl2 (1.5 mM), 6.25% of Dimethyl sulfoxide, 0.4 U of Taq-Gold DNA polymerase, and 10× buffer (AmpliTaq DNA polymerase, Applied Biosystem Inc.). Cycling conditions were: 1 cycle, 7 min at 94 °C; 30 cycles, 60 s at 95 °C, 40 s at 55 °C, and 90 s at 72 °C; and 1 cycle, 5 min at 72 °C. 1 μL polymerase-chain-reaction (PCR) product was mixed with 9 μL of HiDi formamide (Applied Biosystems, Inc.) and 0.3 μL Liz Size Standard (Applied Biosystems, Inc.) and heated at 95 °C for 2 min. The samples were run on an ABI 3500 Genetic Analyzer T (Applied Biosystems, Inc.). PCR products and internal standards were detected using filter set D. Raw data were analyzed with GeneMapper v4.0 software (Applied Biosystems, Inc.).

### 2.7. pEGFP-C1-NPM1 Plasmid Constructs, Cell Transfection and Immunofluorescence Analysis

Generation of the specific plasmids and constructs cloning were commissioned to GeneScript (Piscataway, NJ, USA) once provided with the plasmid pEGFP-C1-NPM1wt as a template and sequence information. For ectopic expression of the GFP-*NPM1* fusion proteins, the murine fibroblasts cell line NIH-3T3 were transiently transfected with plasmids: p-EGFP-C1 empty vector, p-EGFP-C1-NPM1wt and p-EGFP-C1-NPM1mutA, as controls, and the p-EGFP-C1-NPM1_new mutant. The subcellular localization of the GFP-*NPM1* fusion proteins was analyzed by confocal microscopy [13,25]. Treatment with the specific exportin-1/XPO1 inhibitor leptomycin B (20 ng/mL for 5 h) (Merck Biosciences Ltd.,Darmstadt, Germany) of NIH-3T3 overexpressing the new GFP-*NPM1* fusion protein was used to evaluate the NES dependence of its subcellular localization, as previously reported [13,25]. For confocal microscopy studies, transfected NIH-3T3 cells, grown on glass coverslips, were rinsed in phosphate-buffered saline (PBS) and fixed in 4% paraformaldehyde, pH 7.4 (10 min), air dried and flipped onto standard glass slides with Mowiol mounting medium (Sigma-Aldrich, St. Louis, MO, USA). Nuclei were stained with 4,6-diamidino-2-phenylindole (DAPI) fluorescent stain in Prolong Gold mounting reagent (Molecular Probes by Life Technologies, Carlsbad, CA, USA) following standard procedures. Confocal analysis was done with a Zeiss LSM 800 confocal microscope (Carl Zeiss, Jena, Germany) using 488 nm (for eGFP) laser line for excitation.

### 2.8. Real-Time Quantitative Polymerase Chain Reaction (RTq-PCR) for NPM1 Mutation A

RTq-PCR for *NPM1* mutation A was performed using the commercially available ipsogen*^®^ NPM1* mutA MutaQuant*^®^* kit (#677513; QIAGEN GmbH, Hilden, Germany), according to the manufacturer’s instructions. An endogenous control (transcript from Abelson, ABL) is amplified from the sample together with the *NPM1* mutA transcript. Standard curves of known amounts of both the endogenous ABL control and *NPM1* mutA cDNA allow the calculation of the ratio of *NPM1* mutA signal to endogenous ABL signal in each sample. The kit allows quantification of *NPM1* mutA transcripts for minimal residual disease (MRD) monitoring. Notably, the specificity of the kit is based on the use of specific reverse primers for the *NPM1*mutA allele.

## 3. Results

### 3.1. Identification of a New NPM1 Mutation Involving Exon 12

In our previously reported study [17], 387 out of the 929 bone marrow samples analyzed (41.6%) showed cytoplasmic positivity for *NPM1* (NPMc^+^) at IHC (Figure 1). Five out of 387 (1.3%) NPMc^+^ AML cases harbored rare *NPM1* non-exon 12 mutations, specifically in exon 9 (*n* = 1), exon 11 (*n* = 1) and exon 5 (*n* = 3) [17] (Discrepancy 1, Figure 1). Of course, these rare non-exon 12 mutants were not recognized at WB analysis by the anti-*NPM1* mutant antibody that was raised against the C-terminus of mutant A [20]. Instead, all cases with mutation in exon 12, but one, were recognized by the anti-*NPM1* mutant antibody at WB confirming the value of this methodological approach in identifying at least the great majority of *NPM1* mutant proteins in AML samples and in predicting the underlying mutation in *NPM1* exon 12 [20] (Discrepancy 2, Figure 1).

**Figure 2:**
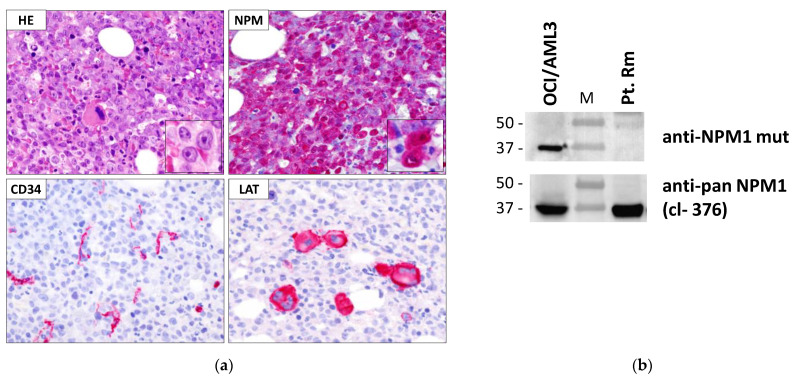
(**a**) Immunohistochemical staining of bone marrow trephine from pt. Rm. HE: hematoxylin-eosin staining; NPM: mouse monoclonal anti-pan*NPM1*, clone 376; CD34: anti-CD34 mAb (to note, blood vessels in tissue sections are used as positive control for CD34 staining). LAT: linker for activation of T cell (marker for megakaryocytes); APAAP technique; hematoxylin counterstaining. Images were collected using an Olympus B61 microscope with a UPlanApo 40×/0.85U and UPlan FI 100×/1.3 NA oil objective for the insets; Camedia 4040, Dp_soft Version 3.2; and Adobe Photoshop CC 2019; (**b**) WB analysis of total protein extracts from pt. Rm. OCI/AML3: positive control for *NPM1* mutant A expression. M: molecular weight marker.

The single case not recognized by WB, hereinafter referred to as pt. Rm (patient sample from Rome, Italy) (Figure 2), was further investigated. IHC analysis on pt. Rm bone marrow biopsy showed diffuse infiltration by leukemic blasts with evident positivity at cytoplasmic level for *NPM1* (*NPM1* (Figure 2a). Myelo-monocytic blasts appeared negative for CD34 (Figure 2a), as typically observed in *NPM1*-mutated AML [1]. Additionally, Bcl-2 protein appeared overexpressed (not shown), as observed in the great majority of AML at diagnosis. Dysplastic megakaryocytes were detected in clusters (Figure 2a). As mentioned, WB analysis with anti-*NPM1* mutant antibody was negative (Figure 2b).

Interestingly, Sanger and deep targeted sequencing of *NPM1* performed on the genomic DNA extracted from pt. Rm peripheral blood (PB) showed the insertion/duplication of the 4 nucleotides (nt) TCTG characterizing the most common *NPM1* mutation type A (NM_002520.6:c.860_863dupTCTG) [1], that, however, was accompanied (15 nucleotides downstream) by a deletion of eight nucleotides (NM_002520.6:c.879_*1delTCTTTAAG), a composite genetic event not previously described (Supplementary Table S1). Specifically, the variant allelic frequencies (VAFs) for the 4-nt TCTG insertion/duplication and the new 8-nt deletion were 16% and 20.5%, respectively, in keeping with the about 40% AML blasts representation in the analyzed patient’s sample (considering that *NPM1* mutation are heterozygous) (Table 1).

**Figure 3:**
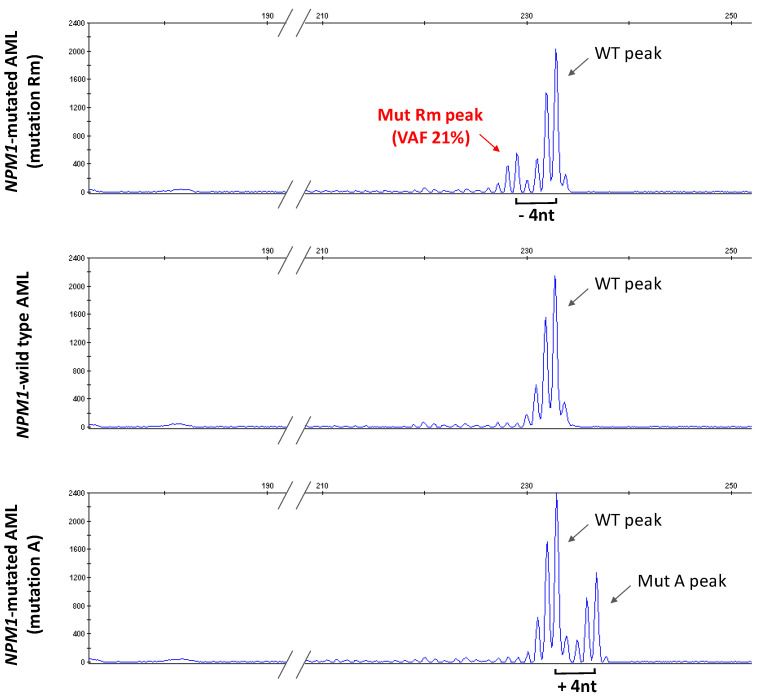
Genomic DNA *NPM1* fragment analysis. Electropherograms of genomic DNA fragments of *NPM1* exon 12 extracted from PB of the AML patient Rm (upper panel), AML sample with wild type *NPM1* (middle panel) and AML sample with *NPM1* mutation A (lower panel). Peaks corresponding to WT (wild type), mutant A (Mut A) and mutant Rm (Mut Rm) are indicated. Fragments from Mut Rm are 4 nucleotides shorter (−4 nt) than WT. Fragments from Mut A are 4 nucleotides longer (+4 nt) than WT. The variant allelic frequency (VAF) of the Rm mutated allele is shown.

To confirm that the two events (4-nt insertion and 8-nt deletion) occurred in the same allele of *NPM1* gene, we performed genomic DNA fragment analysis for *NPM1* exon 12 of the pt. Rm sample in parallel with either wild type or mutation A control (Figure 3). As expected, in the pt. Rm sample, the electropherogram revealed two fragment peaks, one of the same size of the wild type, and the other 4 nt shorter, being the result of the composite 4 nt insertion/8 nt deletion. No peaks of other sizes, and in particular no peak corresponding to mutant A, were evident. These data prove that indeed the 4 nt TCTG insertion/duplication and the new 8 nt deletion occurred in the same allele of *NPM1* gene. Quantitation of the mutation Rm peak indicated a 21% VAF, in line with the values obtained by deep targeted sequencing of *NPM1* (Figure 3, Supplementary Table S1).

**Figure 4:**
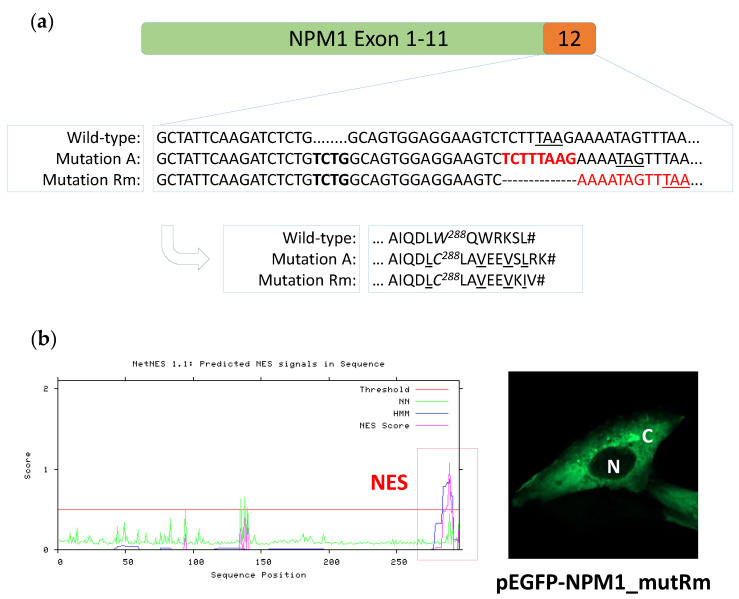
The new *NPM1* mutation Rm disrupts the NoLS and inserts a C-terminal NES. (**a**) Nucleotide sequences of exon 12 in *NPM1* wild-type, mutation A and new composite mutation Rm, and corresponding predicted protein sequences (arrow). The 4 nucleotide insertion TCTG in mutation A and mutation Rm is highlighted in bold black. The 8 nucleotides lost in composite mutation Rm are highlighted in bold red. The C-terminal NES motifs in mutant A and mutant Rm are underlined. (**b**) Left, NES motif prediction in the new *NPM1* mutant Rm. The newly acquired C-terminal NES is highlighted in the red box. Right, representative image of NIH-3T3 overexpressing the new GFP-*NPM1* mutant Rm fusion protein. Images were acquired using a Zeiss LSM 800 confocal microscope (Carl Zeiss) with a 488-nm (for eGFP) laser line for excitation, and a 63×/1.4 OIL Plan-Apochromat objective. N: nucleus; C: cytoplasm.

Occurring on the same *NPM1* allele, the 8 nt frame-shifting deletion, which on its own would just replace the last protein amino acid (L294K; Supplementary Table S1), when combined with the preceding mutation A (4 nt TCTG insertion) leads to elimination of the ‘planned’ stop codon typical of mutation A (W288CfsTer12) and introduces an earlier ‘new’ stop codon (W288CfsTer11; Figure 4a).

### 3.2. The New NPM1 Mutant Protein Displays a New C-Terminal Nuclear Export Signal (NES) Motif

We then analyzed the consequences of this new composite mutation at the protein level. The two events described above result in a new mutant *NPM1* protein (W288CfsTer11), here named Rm, 297 amino acid long, with a C-terminal sequence differing from the mutant A only for the last amino acids (mutRm_^288^CLAVEEVKIV versus mutA_^288^CLAVEEVSLRK) (Figure 4a). In particular, protein sequence prediction indicates that, as for *NPM1* mutant A, the new *NPM1* mutant Rm loses the nucleolar localization signal (NoLS), with the loss, in particular, of both tryptophans 288 and 290 typical of the wild-type *NPM1* protein, whilst it acquires a theoretical new NES. Indeed, a search for acquisition of a NES motif in the new mutant protein sequence, performed by applying the NES detection NetNES 1.1 (http://www.cbs.dtu.dk/services/NetNES, accessed date: 17 August 2021), identified a C-terminal NES with high score (LVVI) (Figure 4b, left). To confirm its predicted cytoplasmic localization, the Rm mutant was expressed as GFP-*NPM1* fusion protein in NIH-3T3 cells and its subcellular localization analyzed at confocal microscopy. As expected by the newly acquired C-terminal NES and the concomitant loss of NoLS, the Rm mutant is localized in the cytoplasm (Figure 4b, right).

### 3.3. The New NPM1 Mutant Protein Locates in the Cytoplasm in a NES-Dependent Manner

**Figure 5:**
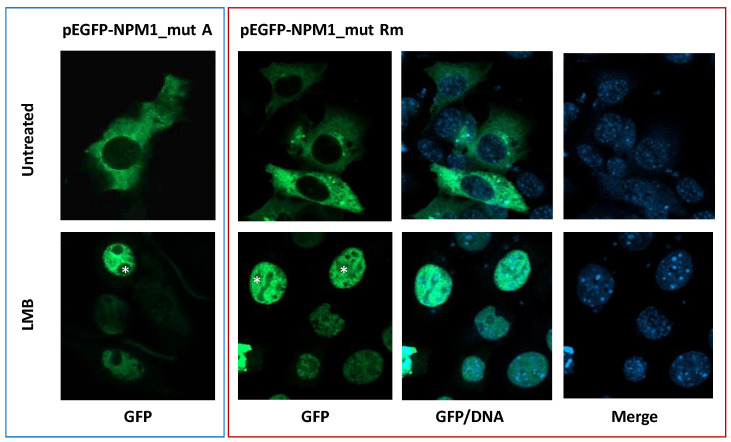
The subcellular localization of the new *NPM1* mutant Rm is NES-dependent. NIH-3T3 overexpressing either the GFP-*NPM1* mutant A or the new GFP-*NPM1* mutant Rm fusion proteins. Untreated: vehicle. LMB: leptomycin B. GFP: green fluorescence protein (of the GFP-*NPM1* fusion protein). DNA: indicates stained nuclei (blue). Images were acquired using a Zeiss LSM 800 confocal microscope (Carl Zeiss) with a 488-nm (for eGFP) laser line for excitation, and a 63×/1.4 OIL Plan-Apochromat objective. Asterisk: nucleoli spaces.

To demonstrate that the Rm mutant locates in the cytoplasm in a NES-dependent manner, its subcellular localization, as a GFP-*NPM1* fusion protein in NIH-3T3 cells, was analyzed at confocal microscopy, either in presence or absence of the exportin-1(XPO1) inhibitor leptomycin B. Whilst in untreated cells, green fluorescence was detected only in the cytoplasm, relocation into the nucleoplasm was observed upon leptomycin B treatment, confirming it is a NES-dependent phenomenon (Figure 5). To note, as expected in NIH-3T3 overexpression system, upon relocation of the Rm mutant into the nucleus, nucleoli are spared most likely due to the loss of NoLS, as happens with *NPM1* mutant A (Figure 5).

### 3.4. Detection of NPM1 Mutant Rm Transcripts by q-RT-PCR for NPM1 Mutation A

Since, as described above, *NPM1* mutation Rm is characterized by the insertion/duplication of the 4 nucleotides TCTG typical of *NPM1* mutation type A (i.e., NM_002520.6:c.860_863dupTCTG) with conservation of the following 15 nucleotides sequence before the eight nucleotide deletion (i.e., NM_002520.6:c.879_*1delTCTTTAAG), we hypothesized that it was identifiable applying the q-RT-PCR assay designed specifically for mutation A and used for diagnostic purposes and minimal residual disease (MRD) monitoring. Indeed, *NPM1* mutant Rm transcripts could be detected and quantified (*NPM1*mutRm = 506 copies/100 Abl) as it occurs for the classical *NPM1* mutation A (Table 1).

### 3.5. Characteristics of AML Carrying NPM1 Mutation Rm

We then analyzed whether patient carrying *NPM1* mutation Rm displayed characteristics typical of the most common AML with *NPM1* mutation A. Besides *NPM1* cytoplasmic positivity at IHC, other biological characteristics in common with other *NPM1*-mutated AML include de novo occurrence, hyperleukocytosis, CD34-negativity, morphological pattern myelo-monocytic phenotype, increased dysplastic megakaryocytes [26,27,28], and normal karyotype [29] (Table 1). Among other genes frequently mutated in *NPM1*-mutated AML, FLT3 was wild-type in pt. Rm, as well as epigenetic genes DNMT3A and IDH1/2, whilst TET2 resulted as mutated with a variant allelic frequency, VAF of 47.2%. Other co-occurring mutations included mutations in SRSF2 (VAF, 50.2%) and ASXL1 (at lower VAF, 8.7%), frequently found in elderly patients [30] (Supplementary Table S2). The VAF values for either *TET2* or *SRSF2* mutations, much higher than *NPM1* mutations (VAF 16/20.5%) and involving almost the totality of the sample cells, suggest *NPM1* mutations arose on a background of a TET2/SRSF2-positive disease.

## 4. Discussion

Here, we report the description of a new mutation involving exon 12 of *NPM1* gene in a patient with diagnosis of AML. As compared to the other variants of exon 12 mutations, this appears peculiar since it introduces the same 4 nucleotides insertion (i.e., TCTG) as mutation A but, due to an accompanying downstream eight-nucleotide deletion, the last amino acids are different.

Nevertheless, as it happens for all *NPM1* mutations, a newly acquired functional C-terminal NES mediated the cytoplasmic accumulation of the new composite mutant.

Whether these two different genetic events occurred simultaneously or independently can be a matter of speculation, given that the two events are separated by 15 nucleotides and the mechanism hypothesized as underlying the four-nucleotide insertion identical to mutation A (i.e., illegitimate activity of the terminal deoxynucleotidyl transferase [31]) is unlikely to explain the following 8-nucleotide deletion. The fact that allele frequency for the eight-nucleotide deletion resulted, upon molecularly barcoded deep sequencing, somewhat higher than the upstream four-nucleotide insertion (20.5% versus 16.1%, respectively) (Supplementary Table S2) might lead to the hypothesis that the eight-nucleotide deletion developed first. However, we can not exclude that, although VAF estimate based on barcoded deep sequencing are generally very accurately determined, the slightly higher VAF of the deletion (4%) could be due to bioinformatics parameters of the variant calling pipeline.

Nevertheless, it would be interesting to dissect in proper experimental models the functional consequences of the 8 nt deletion on its own and the new composite mutation.

The discovery of this new composite mutation involving *NPM1* exon 12 has been made by the application of different diagnostic assays, i.e., immunohistochemistry, molecular analysis (PCR, DNA fragments analysis, deep targeted NGS) and Western blotting, which, more than alternatives, could be considered complementary techniques for an accurate diagnosis [32]. Strikingly, despite a very similar C-terminal protein sequence as mutant A, this mutant was not identified by the anti-*NPM1* mutant A antibody (able to recognize almost all the *NPM1* exon 12 mutants) at WB, suggesting the last amino acids of *NPM1* mutant A (i.e., ^295^SLRK) are important for the antibody reactivity. On the other hand, in contrast to what observed with all the other known exon 12 mutations, the *NPM1* mutant Rm transcripts are detectable and quantifiable by qRT-PCR designed for *NPM1* mutation A (Table 1), suggesting this assay can be used for MRD monitoring in case of *NPM1* mutation Rm.

Biologically, this report reinforces the concept of *NPM1* as ‘born-to be-exported’ in *NPM1*-mutated AML, meaning that only those mutations that lead to exportin-1(XPO1)/*NPM1* mutant interaction and the following dislocation of *NPM1* in the cell cytoplasm are selected to develop leukemia [13,17,18].

In summary, this case underlies on one side the biologic significance of the event ‘cytoplasmic positivity of *NPM1*′ as critical for leukemogenesis in AML, and on the other side the value of using several different techniques in routine diagnostics in order to recognize rare cases leading to similar alterations and define the strategy for MRD monitoring [17,21,32].

## Figures and Tables

**Table 1:** Biological and clinical features of AML patient with *NPM1* mutation Rm.

Characteristics	Pt. Rm
Gender	Male
Age	77
PLT/mmc	36,000
WBC/mmc	102,000
Diagnosis	AML de novo
Blasts % (PB)	40 *
Karyotype	NK
IHC	NPMc^+^
WB (anti-*NPM1*mutA)	negative
*NPM1* mutant transcripts copy n. (MRD assay)	506 copies/100 *Abl*
Dysplastic features	yes (MK)
Blasts CD34 expression	negative
*FLT3*	wild-type
*DNMT3A*	wild-type
*IDH1/2*	wild-type
*TET2*	p.Glu796ArgfsTer15
*SRSF2*	p.Pro95Arg
*ASXL1*	p.Ser1166LysfsTer11
Treatment	Decitabine
Outcome	disease progression/death

PLT, platelets; WBC, white blood cells; IHC immunohistochemistry; NK, normal karyotype; MK, megakaryocytes; * other cells were represented by neutrophils 38%, mature monocytes 15%, lymphocyte 7%.

## Data Availability

For original data, please contact maria.martelli@unipg.it.

## Supplementary Materials

The following are available online at https://www.mdpi.com/article/10.3390/genes12091426/s1, Table S1: Genes subjected to targeted deep sequencing (QIAseq Targeted DNA Custom panel-QIAGEN (CDHS-31667Z-1041), Table S2: List of somatic mutations by barcoded-targeted NGS of myeloid recurrent genes in peripheral blood of AML patient Rm.

## References

[1] Falini B., Mecucci C., Tiacci E., Alcalay M., Rosati R., Pasqualucci L., La Starza R., Diverio D., Colombo E., Santucci A. (2005). Cytoplasmic nucleophosmin in acute myelogenous leukemia with a normal karyotype. N. Engl. J. Med..

[2] Falini B., Nicoletti I., Martelli M.F., Mecucci C. (2007). Acute myeloid leukemia carrying cytoplasmic/mutated nucleophosmin (NPMc^+^ AML): Biologic and clinical features. Blood.

[3] Arber D.A., Orazi A., Hasserjian R., Thiele J., Borowitz M.J., Le Beau M.M., Bloomfield C.D., Cazzola M., Vardiman J.W. (2016). The 2016 revision to the World Health Organization classification of myeloid neoplasms and acute leukemia. Blood.

[4] Grisendi S., Mecucci C., Falini B., Pandolfi P.P. (2006). Nucleophosmin and cancer. Nat. Rev. Cancer.

[5] Grisendi S., Bernardi R., Rossi M., Cheng K., Khandker L., Manova K., Pandolfi P.P. (2005). Role of nucleophosmin in embryonic development and tumorigenesis. Nature.

[6] Boulon S., Westman B.J., Hutten S., Boisvert F.M., Lamond A.I. (2010). The nucleolus under stress. Mol. Cell.

[7] Falini B., Martelli M.P., Mecucci C., Liso A., Bolli N., Bigerna B., Pucciarini A., Pileri S., Meloni G., Martelli M.F. (2008). Cytoplasmic mutated nucleophosmin is stable in primary leukemic cells and in a xenotransplant model of NPMc^+^ acute myeloid leukemia in SCID mice. Haematologica.

[8] McKerrell T., Park N., Moreno T., Grove C.S., Ponstingl H., Stephens J., Crawley C., Craig J., Scott M.A., Hodkinson C. (2015). Leukemia-associated somatic mutations drive distinct patterns of age-related clonal hemopoiesis. Cell Rep..

[9] Falini B., Sportoletti P. (2017). A scale of bad co-mutations in NPM1-driven AML. Blood.

[10] Falini B., Martelli M.P. (2011). NPM1-mutated AML: Targeting by disassembling. Blood.

[11] Hingorani K., Szebeni A., Olson M.O. (2000). Mapping the functional domains of nucleolar protein B23. J. Biol. Chem..

[12] Wang W., Budhu A., Forgues M., Wang X.W. (2005). Temporal and spatial control of nucleophosmin by the Ran-Crm1 complex in centrosome duplication. Nat. Cell Biol..

[13] Bolli N., Nicoletti I., De Marco M.F., Bigerna B., Pucciarini A., Mannucci R., Martelli M.P., Liso A., Mecucci C., Fabbiano F. (2007). Born to be exported: COOH-terminal nuclear export signals of different strength ensure cytoplasmic accumulation of nucleophosmin leukemic mutants. Cancer Res..

[14] Falini B., Albiero E., Bolli N., De Marco M.F., Madeo D., Martelli M., Nicoletti I., Rodeghiero F. (2007). Aberrant cytoplasmic expression of C-terminal-truncated NPM leukaemic mutant is dictated by tryptophans loss and a new NES motif. Leukemia.

[15] Mariano A.R., Colombo E., Luzi L., Martinelli P., Volorio S., Bernard L., Meani N., Bergomas R., Alcalay M., Pelicci P.G. (2006). Cytoplasmic localization of NPM in myeloid leukemias is dictated by gain-of-function mutations that create a functional nuclear export signal. Oncogene.

[16] Albiero E., Madeo D., Bolli N., Giaretta I., Bona E.D., Martelli M.F., Nicoletti I., Rodeghiero F., Falini B. (2007). Identification and functional characterization of a cytoplasmic nucleophosmin leukaemic mutant generated by a novel exon-11 NPM1 mutation. Leukemia.

[17] Martelli M.P., Rossi R., Venanzi A., Meggendorfer M., Perriello V.M., Martino G., Spinelli O., Ciurnelli R., Varasano E., Brunetti L. (2021). Novel Npm1 Exon 5 Mutations and Gene Fusions Leading to Aberrant Cytoplasmic Nucleophosmin in Aml. Blood.

[18] Brunetti L., Gundry M.C., Sorcini D., Guzman A.G., Huang Y.H., Ramabadran R., Gionfriddo I., Mezzasoma F., Milano F., Nabet B. (2018). Mutant NPM1 Maintains the Leukemic State through HOX Expression. Cancer Cell.

[19] Falini B., Martelli M.P., Bolli N., Bonasso R., Ghia E., Pallotta M.T., Diverio D., Nicoletti I., Pacini R., Tabarrini A. (2006). Immunohistochemistry predicts nucleophosmin (NPM) mutations in acute myeloid leukemia. Blood.

[20] Martelli M.P., Manes N., Liso A., Pettirossi V., Verducci Galletti B., Bigerna B., Pucciarini A., De Marco M.F., Pallotta M.T., Bolli N. (2008). A western blot assay for detecting mutant nucleophosmin (NPM1) proteins in acute myeloid leukaemia. Leukemia.

[21] Falini B., Brunetti L., Martelli M.P. (2021). How I diagnose and treat NPM1-mutated AML. Blood.

[22] Quentmeier H., Martelli M.P., Dirks W.G., Bolli N., Liso A., Macleod R.A., Nicoletti I., Mannucci R., Pucciarini A., Bigerna B. (2005). Cell line OCI/AML3 bears exon-12 NPM gene mutation-A and cytoplasmic expression of nucleophosmin. Leukemia.

[23] Tiacci E., Spanhol-Rosseto A., Martelli M.P., Pasqualucci L., Quentmeier H., Grossmann V., Drexler H.G., Falini B. (2012). The NPM1 wild-type OCI-AML2 and the NPM1-mutated OCI-AML3 cell lines carry DNMT3A mutations. Leukemia.

[24] Xu C., Gu X., Padmanabhan R., Wu Z., Peng Q., DiCarlo J., Wang Y. (2019). smCounter2: An accurate low-frequency variant caller for targeted sequencing data with unique molecular identifiers. Bioinformatics.

[25] Falini B., Bolli N., Shan J., Martelli M.P., Liso A., Pucciarini A., Bigerna B., Pasqualucci L., Mannucci R., Rosati R. (2006). Both carboxy-terminus NES motif and mutated tryptophan(s) are crucial for aberrant nuclear export of nucleophosmin leukemic mutants in NPMc^+^ AML. Blood.

[26] Pasqualucci L., Liso A., Martelli M.P., Bolli N., Pacini R., Tabarrini A., Carini M., Bigerna B., Pucciarini A., Mannucci R. (2006). Mutated nucleophosmin detects clonal multilineage involvement in acute myeloid leukemia: Impact on WHO classification. Blood.

[27] Falini B., Macijewski K., Weiss T., Bacher U., Schnittger S., Kern W., Kohlmann A., Klein H.U., Vignetti M., Piciocchi A. (2010). Multilineage dysplasia has no impact on biologic, clinicopathologic, and prognostic features of AML with mutated nucleophosmin (NPM1). Blood.

[28] Sportoletti P., Varasano E., Rossi R., Bereshchenko O., Cecchini D., Gionfriddo I., Bolli N., Tiacci E., Intermesoli T., Zanghi P. (2013). The human NPM1 mutation A perturbs megakaryopoiesis in a conditional mouse model. Blood.

[29] (2013). Genomic and epigenomic landscapes of adult de novo acute myeloid leukemia. N. Engl. J. Med..

[30] Eisfeld A.K., Kohlschmidt J., Mrozek K., Blachly J.S., Walker C.J., Nicolet D., Orwick S., Maharry S.E., Carroll A.J., Stone R.M. (2018). Mutation patterns identify adult patients with de novo acute myeloid leukemia aged 60 years or older who respond favorably to standard chemotherapy: An analysis of Alliance studies. Leukemia.

[31] Borrow J., Dyer S.A., Akiki S., Griffiths M.J. (2019). Molecular roulette: Nucleophosmin mutations in AML are orchestrated through N-nucleotide addition by TdT. Blood.

[32] Falini B., Martelli M.P., Pileri S.A., Mecucci C. (2010). Molecular and alternative methods for diagnosis of acute myeloid leukemia with mutated NPM1: Flexibility may help. Haematologica.

