# Supplementary material for: A Curious Novel Combination of Nucleophosmin (NPM1) Gene Mutations Leading to Aberrant Cytoplasmic Dislocation of NPM1 in Acute Myeloid Leukemia (AML)
Source: Genes (Basel). 2021 Sep 16;12(9):1426. doi: 10.3390/genes12091426 (PMC8468273; doi:10.3390/genes12091426)
Supplement: Supplementary file 1 [file genes-12-01426-s001.zip › genes-1369053-supplementary.pdf]

Table S1. Genes subjected to targeted deep sequencing

(QIAseq Targeted DNA Custom panel-QIAGEN (CDHS-31667Z-1041)

Gene	Target (exon)	Gene	Target (exon)	Gene	Target (exon)	Gene	Target (exon)
**ANKRD26**	5’utr	**ETV6/TEL**	1-8	**KRAS**	2, 3	**SF3B1**	13-16
**ASXL1**	12	**EZH2**	All exons	**MBD4**	1-8	**SMC1A**	2, 11, 16, 17
**BCOR**	All exons	**FLT3**	14,15,20	**MPL**	10	**SRSF2**	1
**BCORL1**	All exons	**GATA1**	2	**NPM1**	12	**STAG2**	All exons
**BRAF**	15	**GATA2**	4-6	**NRAS**	2, 3	**TET2**	3-11
**CALR**	9	**HRAS**	2, 3	**PHF6**	All exons	**TP53**	2-11
**CBL**	8-9	**IDH1**	4	**PPM1D**	5-6	**U2AF1**	2, 6
**CEBPA**	All exons	**IDH2**	4	**PTPN11**	3, 13	**WT1**	7,9
**CSF3R**	10,14-18	**JAK2**	12, 14	**RAD21**	All exons	**ZRSR2**	All exons
**DDX41**	5,17	**JAK3**	13	**RUNX1**	1-6		
**DNMT3A**	All exons	**KIT**	2, 8-11, 13, 17	**SETBP1**	4 (partial)		

Table S2. List of somatic mutations by barcoded-targeted NGS of myeloid recurrent genes in peripheral blood of AML patient Rm.

Gene	Chr	Coordinate	HGVS coding sequence name	HGVS protein sequence name	Variant type	Variant allele frequency (%)	Unique Variant coverage depth
SRSF2	17	74732959	NM_003016.4:c.284C>G	NP_003007.2:p.Pro95Arg	missense	50.2	510
TET2	4	106157477	NM_001127208.2:c.2379_2385delATCAAGC	NP_001120680.1:p.Glu796ArgfsTer15	frameshift	47.2	688
NPM1	5	170837562	NM_002520.6:c.879_*1delTCTTTAAG	NP_002511.1:p.Leu294Lys	frameshift	20.5	108
NPM1	5	170837543	NM_002520.6:c.859_860insTCTG	NP_002511.1:p.Trp288CysfsTer12	frameshift	16.1	80
ASXL1	20	31024010	NM_015338.5:c.3497_3510delGCCCCAGTTCTTTA	NP_056153.2:p.Ser1166LysfsTer11	frameshift	8.7	104
